# Passenger Lymphocyte Syndrome and Liver Transplantation

**DOI:** 10.1155/2008/715769

**Published:** 2009-03-05

**Authors:** Maxime Audet, Fabrizio Panaro, Tullio Piardi, Ping Huang, Murat Cag, Jacques Cinqualbre, Philippe Wolf

**Affiliations:** Centre de Chirurgie Viscérale et de Transplantation, Hôpital de Hautepierre, Hopitaux Universitaires de Strasbourg-Université Louis Pasteur, Avenue Molière, 67200 Strasbourg, France

## Abstract

The authors reviewed the passenger lymphocyte syndrome (PLS) that has appeared after transplantation. The definition, mechanism, serological, clinical features, and treatment for PLS after solid organ transplantation, especially liver transplantation, are described. The PLS refers to the clinical phenomenon of alloimmune hemolysis resulting from the adoptive transfer of viable lymphocytes from donor during solid organ or hematopoietic stem cell transplant. Sometimes, it is very severe and may cause “unexplained” hemolysis during the postoperative period. The authors reviewed literature about the PLS in liver transplantation.

## 1. INTRODUCTION

The passenger lymphocyte syndrome is due to the production of antibodies by the donor B lymphocytes “passenger lymphocytes” in a primary or secondary immune response against the recipient's red blood cell antigens [1]. 

PLS is mostly often seen in solid organ transplantation with a minor ABO mismatch [1]. The factors that affect the severity of hemolysis including the quantity of transplanted lymphoid tissue are: the level of red cells isoagglutinins in the donor before transplant and the rapid rise in antibody titer in the recipient after transplant [2]. It usually occurs in 1–3 weeks posttransplant and resolves within 3 months posttransplant, and is a self-limited process [3]. PLS usually results from antibodies active against the ABO and Rh systems. Rarely, it may occur due to non-ABO/Rh antibodies, particularly if the organ donor has been previously sensitized to other red cell antigens by transfusion or pregnancy [4–7]. It has been reported that PLS developed in two of four patients who got organs for a same donor [5]. Hemolysis due to PLS trends to be less common following solid organ transplant [1, 3], and the relative frequency of PLS appears to be related to the volume of transplanted lymphoid tissue. It is more frequent in heart and lung transplants and less in liver and kidney transplants. Only few anecdotal cases are reported in the literature after liver transplantation [1–5]. 

## 2. DEFINITION OF PLS

The appearance of unexpected antibodies of A and B specificity in recipients of kidney allografts from ABO minor mismatched donors was first reported in the early 1980s. Then, more than 100 cases involving liver, kidney, pancreas, spleen, heart, lung, and heart-lung were published in 1991. The source of the isohemagglutinins is viable donor B lymphocytes passively transferred with the organ at the time of transplantation. The phenomenon has been termed the “passenger lymphocyte syndrome.” The donor origin of the antibody has been confirmed using immunoglobulin allotyping [1]. During PLS, the donor memory B lymphocytes produce antibodies against recipient red blood cells causing hemolysis [8]. A fascinating immunologic phenomenon can occur in the setting of a minor ABO mismatch. Viable lymphocytes contaminating the donor can temporarily reside in the recipient, and if they are stimulated shortly after transplant by recipient or transfused red cell antigens, they can start producing antibodies during their life. Leo et al. [9] reported that PLS with severe hemolytic anemia was due to an anti-JK^*α*^ on day 19 after allogeneic peripheral blood progenitor cell transplantation. 

## 3. MECHANISM OF PLS 3: ANTIBODY (AB), ANTIGEN (AG)

Three different groups of ABO incompatibility can be distinguished in transplantation: minor, major, and bidirectional ABO incompatibility. Major ABO-incompatible (e.g., A into O) is characterized by the presence of preformed antidonor A/B Ab directed against donor ABO Ag expressed on transplanted cells. Recipients of minor ABO-incompatible transplantation (e.g., O into A) express ABO Ag that are not expressed in the donor and are at risk for graft-versus-host (GvH) reactions such as delayed hemolysis of recipient red blood cell (RBC) due to PLS. Although major ABO-incompatible organs are not used routinely for transplantation, minor ABO-incompatible organs are frequently used to meet the demand for organs. Bidirectional ABO incompatibility (e.g., A into B) represents a combination of major and minor ABO incompatibility and puts the recipient at risk for both host-versus-graft and GvH [3]. 

Therefore, the PLS can be regarded as a type of graft-versus-host reaction. Most commonly, passenger lymphocyte hemolysis is seen with a minor ABO mismatch, although it can occur with other blood group system mismatches [3–7]. Immunocompetent donor memory B lymphocytes produce antibodies in a secondary immune response against the recipient's red cells. The massive red cells destruction is thought to be complement-mediated [8]. Sokol et al. [3] thought there were three different posttransplant immune-mediated hemolysis which are autoimmune hemolytic anemia, PLS, and major blood mismatch. PLS is most common within the ABO system when there is a minor mismatch between the donor and the recipient, although it can occur within other blood group system, such as Rh, Kidd, and Lewis. Immunosuppression used posttransplantation has an important etiologic role. Cyclosporine and tacrolimus can permit rapid proliferation of spared lymphocyte B cells, which produce antibodies. The antibodies typically become evident at 5 to 17 days after transplant, and are usually undetectable by 3 months. It is rarely fatal. However during autoimmune hemolytic anemia, in allogeneic bone marrow and/or peripheral blood progenitor cell transplants, antibodies are produced by donor lymphocytes against donor-produced red cells. While in solid organ transplants, antibodies are produced by recipient lymphocytes against recipient red cells. Then, during major blood group mismatch, recipient produces antibodies against donor red cells. 

## 4. PLS IN SOLID ORGAN TRANSPLANTATION

### 4.1. Liver transplantation

The incidence of biochemical PLS in liver transplantation is 30%–40%. In fact, in almost all the cases (68–100%) the antibodies are directed against Rh blood group antigens, and only 30–40% of these antibodies led to immune hemolysis [1, 4, 10–13]. 

Several reports have described the appearance of RBC antibodies in patients receiving liver transplants from immunized donors. In almost all of these cases, the antibodies were directed against Rh blood group antigens, and only a few of these antibodies led to moderate-severe immune hemolysis (anti-D, 13 cases, anti-c, 2 cases, and anti-e, 1 case) [4, 11, 12, 14]. 

PLS can develop abruptly and can vary from mild to severe. The severity is mediated by the quantity of transplanted lymphoid tissue, donor titers of isoagglutinins and a rapid rise in antibody titer in the recipient after transplantation [7]. Onset is usually 1–3 weeks posttransplant and is a self-limited process, usually resolving by 3 months posttransplant [13]. 

In particular, Triulzi et al. [14] reported that 56% (five out of nine) of liver transplant recipients developed antibody, and five had biochemical (low haptoglobin, high transaminases, and unconjugated bilirubin) evidence of hemolysis. In Japan, it has been reported that two cases developed hemolytic reaction due to graft-versus-host antibody in living donor liver transplantation [11]. Shortt et al. [4] found that severe hemolysis due to PLS in all three recipients of organs from a single donor with multiple red cell alloantibodies. The liver transplant recipient required augmentation of immunosuppression to treat immune hemolysis due to anti-B, -D, -C, and -Cellano (K). In addition, Grosskreutz et al. [12] described HLA typing was critical diagnosis of the recipient as Evans syndrome, and suggest that recipients of solid organs from donors that are partially matched in the direction of rejection may need to be closely monitored for graft-versus-host disease. 

## 5. OTHER SOLID ORGAN TRANSPLANTATION

Ramsey [15] reported that the frequency of PLS was highest, at 70% in heart-lung transplant recipients, and 17% in kidney transplant recipients. Salerno et al. [6] reported a study of seven minor ABO-incompatible lung transplant recipients identified antibody and hemolysis in 57% (four out of seven) and 29% (two out of seven), respectively. It has been suggested that the amount of lymphoid tissue transplanted with the organ accounts for these differences. In addition, antibodies to red cell antigens outside the ABO system have been reported in association with transplanted kidney, pancreas-kidney, pancreas, liver, and heart-lung [1]. Seltsam et al. [5] found development of donor alloantibodies in two recipients of separate organs (liver and pancreas-kidney) from the same donor. Panaro et al. [7] reported a severe hemolysis due to PLS in a living-related bowel transplant recipient in an ABO-compatible donor-recipient pair (O into A). 

## 6. SEROLOGICAL FEATURES IN PLS

Donor-derived ABO antibody typically develops 7–14 days after liver transplantation. At the same time, direct antiglobulin test is always positive. The serum antibody is predominantly IgG, but it may also be IgM. Passenger lymphocyte derived antibodies are short-lived, persisting for about 2–3 weeks in liver transplant recipients and 5 weeks in kidney transplant recipients [1]. Using flow cytometry and nested polymerase chain reaction can diagnose the donor lymphocyte microchimerism [5]. 

## 7. CLINICAL FEATURES IN PLS

PLS appears mostly in a variable proportion of ABO minor mismatched organ transplant recipients. In liver transplant recipients, from 68% [15] to 100% [14] with donor-derived antibody develop hemolysis. Although the hemolysis is usually mild and self-limited, substantial morbidity such as acute renal failure, disseminated intravascular coagulation, hypotension, and multiorgan failure, has been reported [1, 14–18]. 

## 8. TREATMENT FOR PLS

Today, there are no reliable factors can predict which recipients of ABO minor mismatched organs will develop passenger lymphocyte derived antibodies or PLS. Management strategies for PLS are anecdotal, as described in case reports and small series. Therapeutic interventions include escalation of immunosuppression, immune modulation (e.g., by IVIG) and specifically targeting B-lymphocytes using monoclonal antibodies such as rituximab [7]. 

In most cases PLS can be treated only with transfusions. Transfused red cells should be of organ donor ABO group to replace susceptible red cells with cells that would not be hemolyzed. Plasmapheresis or red cell exchange with donor-type red cells may need to treat those who develop severe hemolysis. Sometimes steroids is effective to treat PLS. Anti-CD20 monoclonal antibody (rituximab) is also effective for the severe patients. 

There has been considerable interest in the past several yearsin the use of the monoclonal antibodies widely used in the treatmentof B-cell lymphoid neoplasms, namely rituximab (Rituxan), and to a lesser extent alemtuzumab (Campath-1H). Furthermore, the role of monoclonal antibodies specifically rituximab, in the therapy of autoimmunecytopenias has been validated recently. However, the experience with the monoclonal antibodies for the PLS in solid organ transplantation is very limited. In fact up-to-date no experience was reported in case of PLS after LT. It is likely thatas our experience with the drug evolves, it will be used atan earlier point in therapy, before more toxic immunosuppressives, rather than only in refractory cases [19, 20]. 

Panaro et al. [7] reported a case of severe hemolysis due to PLS occurred in a 4-year-old blood group A recipient of living donor (blood group O) small-bowel transplantation. The patient was treated successfully with plasmapheresis, blood O transfusion, and rituximab. Sokol et al. [3] reviewed the treatment of hemolysis due to PLS posttransplant including transfusion of compatible red cells and/or plasma components with or without steroids, and maintenance of adequate renal perfusion, plasma exchange and/or exchange transfusion. Sometimes splenectomy should be considered. 

## 9. CONCLUSION

Minor ABO mismatched organ transplants are often used clinically. PLS is a common complication of this practice. PLS can occur in 30%–40% of liver transplant recipients including cadaveric and living donors. Today, no specific treatment is available for the PLS. Therefore, PLS can be treated with transfusion, plasmapheresis, red cell exchange, and anti-CD20 monoclonal antibody. 
